# Supplementary material for: An Orc6 tether mediates ORC binding-site switching during replication origin licensing
Source: Proc Natl Acad Sci U S A. 2025 Oct 7;122(41):e2510685122. doi: 10.1073/pnas.2510685122 (PMC12541460; doi:10.1073/pnas.2510685122)
Supplement: Supplementary file 1 — Appendix 01 (PDF) [file pnas.2510685122.sapp.pdf]

PNAS

Supporting Information for

An Orc6 tether mediates ORC binding site switching during replication origin licensing

David Driscoll[1], Larry J. Friedman[2], Jeff Gelles[2]*, and Stephen P Bell[1]*

[1]Howard Hughes Medical Institute, Department of Biology, Massachusetts Institute of Technology, Cambridge, MA 02139, USA

[2]Department of Biochemistry, Brandeis University, Waltham, MA 02454, USA

Stephen P. Bell
Email : spbell@mit.edu
Phone : 617-253-2054

Jeff Gelles
Email : gelles@brandeis.edu
Phone : 781-736-2377

This PDF file includes:

Figures S1 to S9
Tables S1 to S3
SI Methods

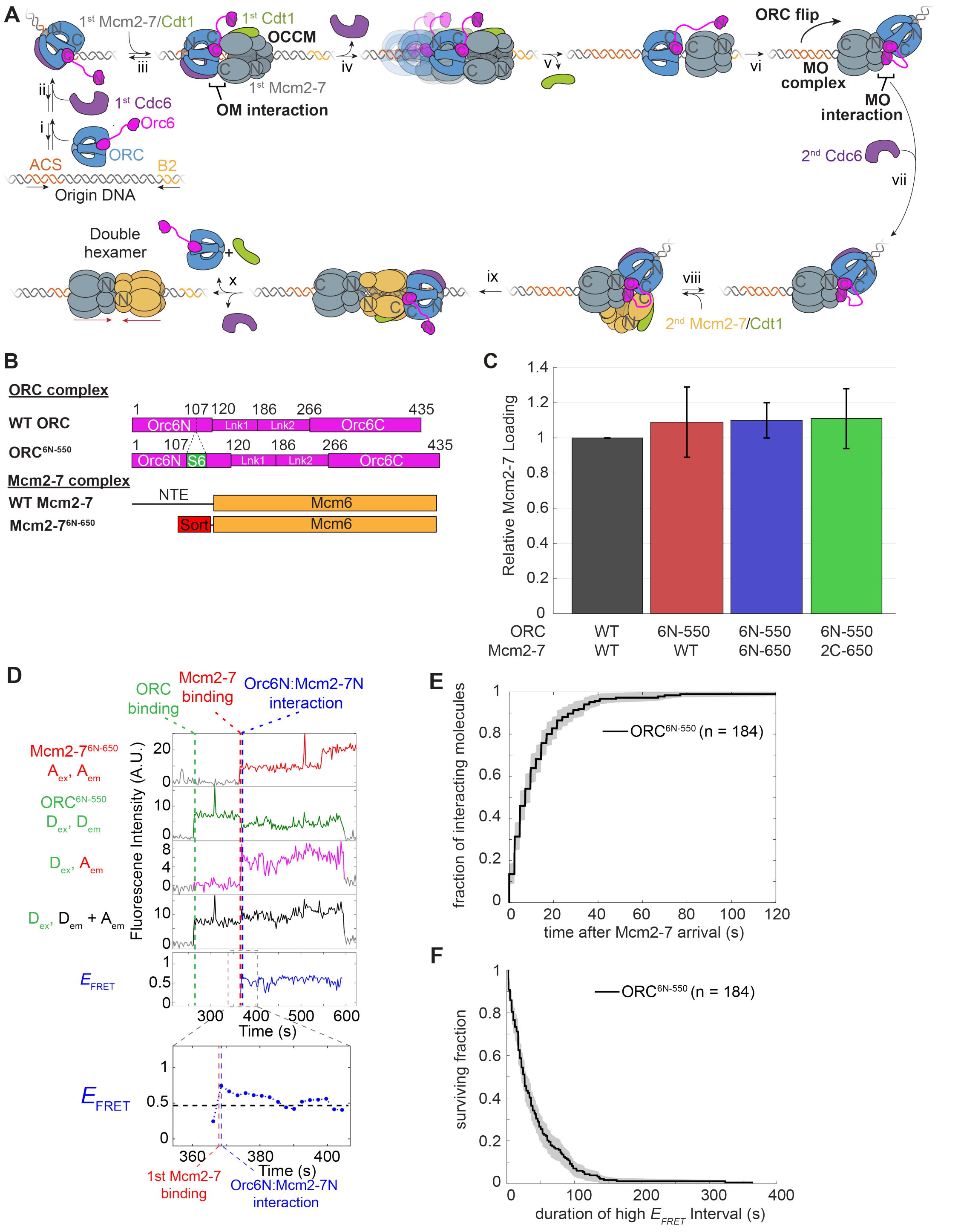

Figure S1: Helicase loading model prior to this study and additional information about assay.

A: Model of helicase loading prior to data from this study. See text for description. N and C notations on ORC and Mcm2-7 represent the N- and C-tiers of each protein. N and C notations on Orc6 represent the N- and C-terminal TFIIB-related domains.

B: Labeling approach for ORC^{6N-550} and Mcm2-7^{6N-650}. ORC^{6N-550} was modified by inserting an S6 peptide tag (GDSLSWLLRLLN) after amino acid 107 in Orc6. ORC containing the modified Orc6 was purified and labeled using Sfp synthase and acetyl-CoA Dylight 550. For Mcm2-7^{6N-650}, Mcm6 was modified by replacing amino acids 1-103 (NTE) with an N-terminal recognition tag for Sortase. Mcm2-7 containing the modified Mcm6 was labeled using Sortase to attach Dylight 650-maleimide conjugated peptide.

C: Ensemble helicase-loading assays were used to assess ORC^{6N-550}, Mcm2-7^{6N-650}, and Mcm2-7^{2C-650} function. SDS-gel band intensities were quantified relative to parallel reactions with unmodified wild-type ORC and Mcm2-7 to obtain relative extents of Mcm2-7 loading. Errors are SEM. N = 2 for each reaction.

D: Additional record showing Mcm2-7^{6N-650} recruitment to ORC^{6N-550} resulting in double hexamer formation plotted as in Figure 1C. This record illustrates that the high E_{FRET} state does not have to be maintained at all times for successful double-hexamer formation.

E: Time to formation of the Orc6N:Mcm2-7N interaction relative to Mcm2-7^{6N-650} arrival. Horizontal axis is time after Mcm2-7^{6N-650} recruitment. Vertical axis is the fraction of stable ORC-Mcm2-7 complexes that formed the Orc6N:Mcm2-7N interaction. Shading represents 95% CI. Only the first Orc6N:Mcm2-7N interaction for a given recruitment of Mcm2-7^{6N-650} by ORC^{6N-550} was considered for this analysis. Molecules that did not achieve a high E_{FRET} state were not included in the analysis.

F: Cumulative survival curve of Orc6N:Mcm2-7N interaction. This plot shows the distribution of lifetimes for the Orc6N-Mcm2-7N interactions. The same subset of events included in E are presented in this analysis. Shading represents 95% CI.

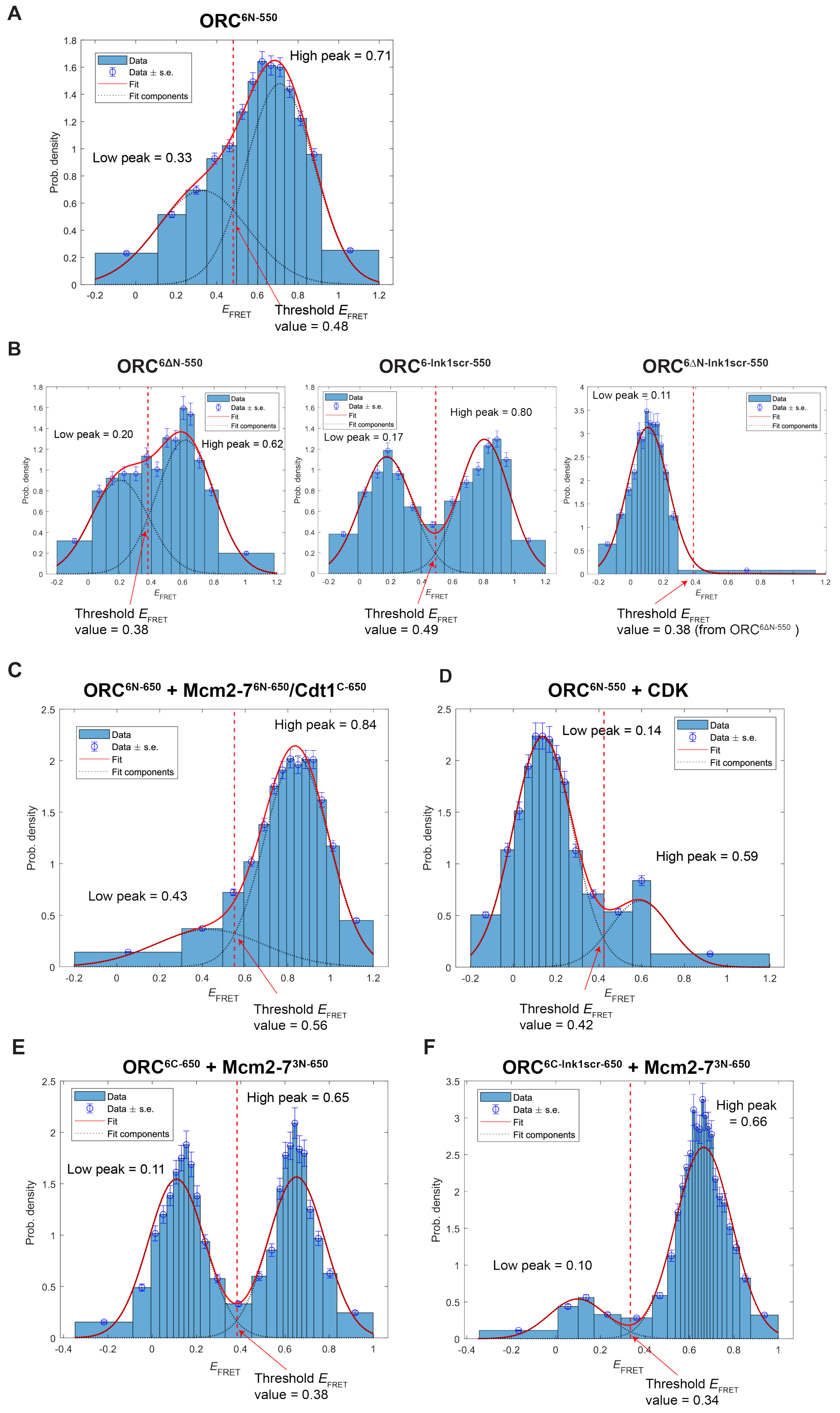

Figure S2: Fits of E_{FRET} distributions to two-component Gaussian mixture models (see Methods). The thresholds to delineate between low and high E_{FRET} states were taken to be the points (arrows) at which the component curves intersect. Fit parameters and N values are presented in Table S1.

A: Experiment (Figure 1) with ORC^{6N-550} and Mcm2-7^{6N-650}.

B: Experiments (Figure 2) with ORC$^{6\Delta N\text{-}550}$ and Mcm2-7$^{6N\text{-}650}$ (left), ORC$^{6\text{-}lnk1scr\text{-}550}$ and Mcm2-7$^{6N\text{-}650}$ (middle), and ORC$^{6\Delta N\text{-}lnk1scr\text{-}550}$ and Mcm2-7$^{6N\text{-}650}$ (right).

C: Experiment (Figure 3) with ORC$^{6N\text{-}550}$ and Mcm2-7$^{6N\text{-}650}$ / Cdt1$^{C\text{-}650}$.

D: Experiment (Figure 4) with ORC$^{6N\text{-}550}$, Mcm2-7$^{6N\text{-}650}$, and CDK.

E: Experiment (Figure 5) with ORC$^{6C\text{-}550}$ and Mcm2-7$^{3N\text{-}650}$.

F: Experiment (Figure 5) with ORC$^{6C\text{-}lnk1scr\text{-}550}$ and Mcm2-7$^{3N\text{-}650}$.

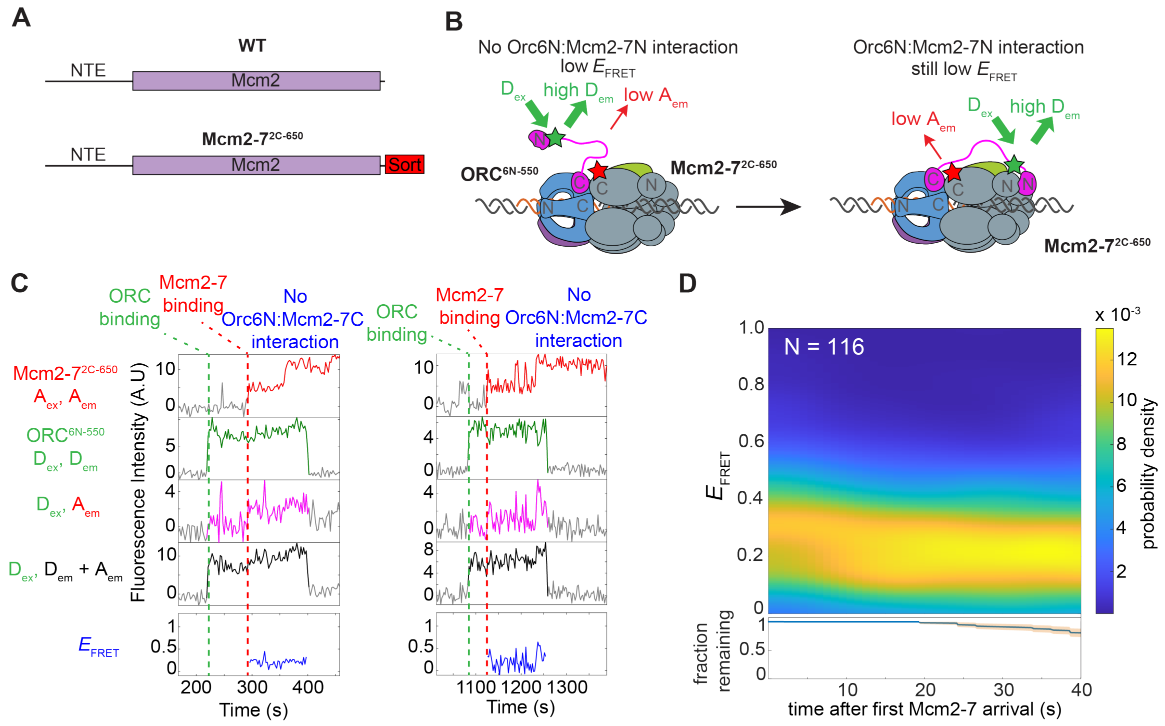

Figure S3: ORC^{6N-550} FRET with Mcm2-7 is specific to the Mcm2-7 N-terminus.

A: Labeling approach for Mcm2-7^{2C-650}. Mcm2 was modified by attaching a C-terminal recognition tag for Sortase. Mcm2-7 complexes with the Sortase tags were coupled to a Dylight-650-labeled peptide using Sortase. See Methods for details.

B: Model of FRET observed in an experiment in which ORC^{6N-550} was labeled with donor fluorophore as in Figure 1 but Mcm2-7^{2C-650} was labeled with an acceptor fluorophore at the C-terminus of Mcm2.

C: Two representative single-DNA records of ORC^{6N-550} recruitment of Mcm2-7^{2C-650}, plotted as in Figure 1C.

D: E_{FRET} distribution heat map for 116 DNA molecules where ORC^{6N-550} recruited Mcm2-7^{2C-650}. Bottom plot shows fraction of ORC^{6N-550}-Mcm2-7^{2C-650} complexes that retain both ORC^{6N-550} and Mcm2-7^{2C-650} bound and the 95% CI are shown in the bottom plot (blue curve, orange shading).

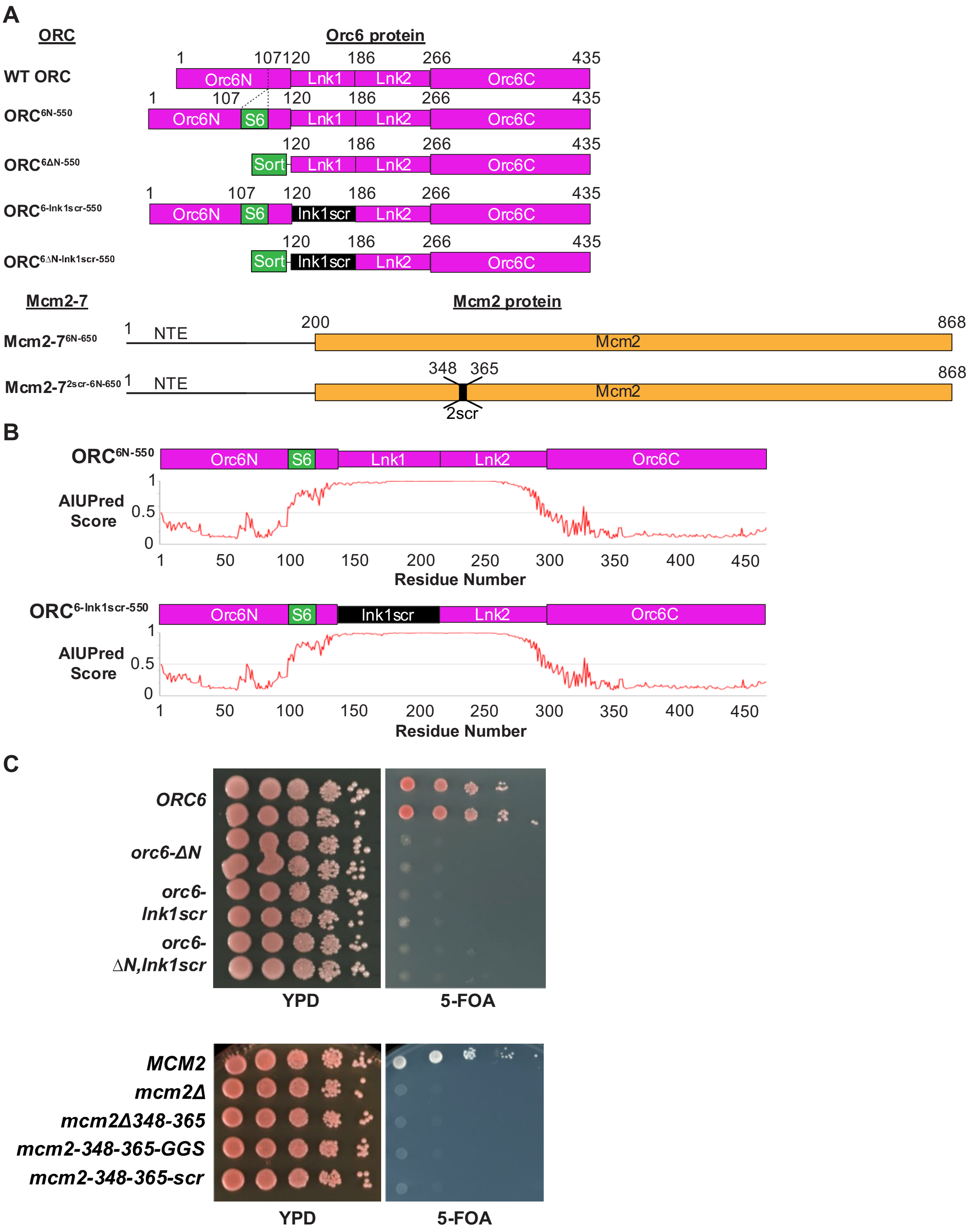

Figure S4: ORC constructs used for Orc6 mutant experiments.

A: Diagram of Orc6 mutants used in Orc6-tether assays. For each ORC complex tested in this study, mutations and the modifications made for labeling of Orc6 protein are shown. $ORC^{6N\text{-}550}$ and $ORC^{6\text{-}Ink1scr\text{-}550}$ were labeled with the Sfp synthase/S6 tag system described

in Figure S1B. $ORC^{6\Delta N\text{-}550}$ and $ORC^{6\Delta N\text{-}Ink1scr\text{-}550}$ were labeled using Sortase as described in Figure S1B. See Methods for protein labeling details.

B: Structure prediction of amino acids in $ORC^{6N\text{-}550}$ and $ORC^{6\text{-}Ink1scr\text{-}550}$ using AIUpred (1). Values greater than 0.5 indicate regions predicted to be disordered.

C: Mcm2 and Orc6 regions required for tether formation are essential. The phenotype of the *mcm2* and *orc6* mutants defective in tether formation were tested for viability using a plasmid-shuffle assay (see Methods). In addition to the scramble mutant tested biochemically (mcm2-348-365scr), we also tested mutants that deleted this region or replaced the same region with GGS residues. Growth on 5-FOA selects against cells containing wild-type *MCM2* or *ORC6* plasmids, revealing the functionality of the remaining mutant allele. Ten-fold serial dilutions of cells were grown on the indicated media for 2 days at 30°C.

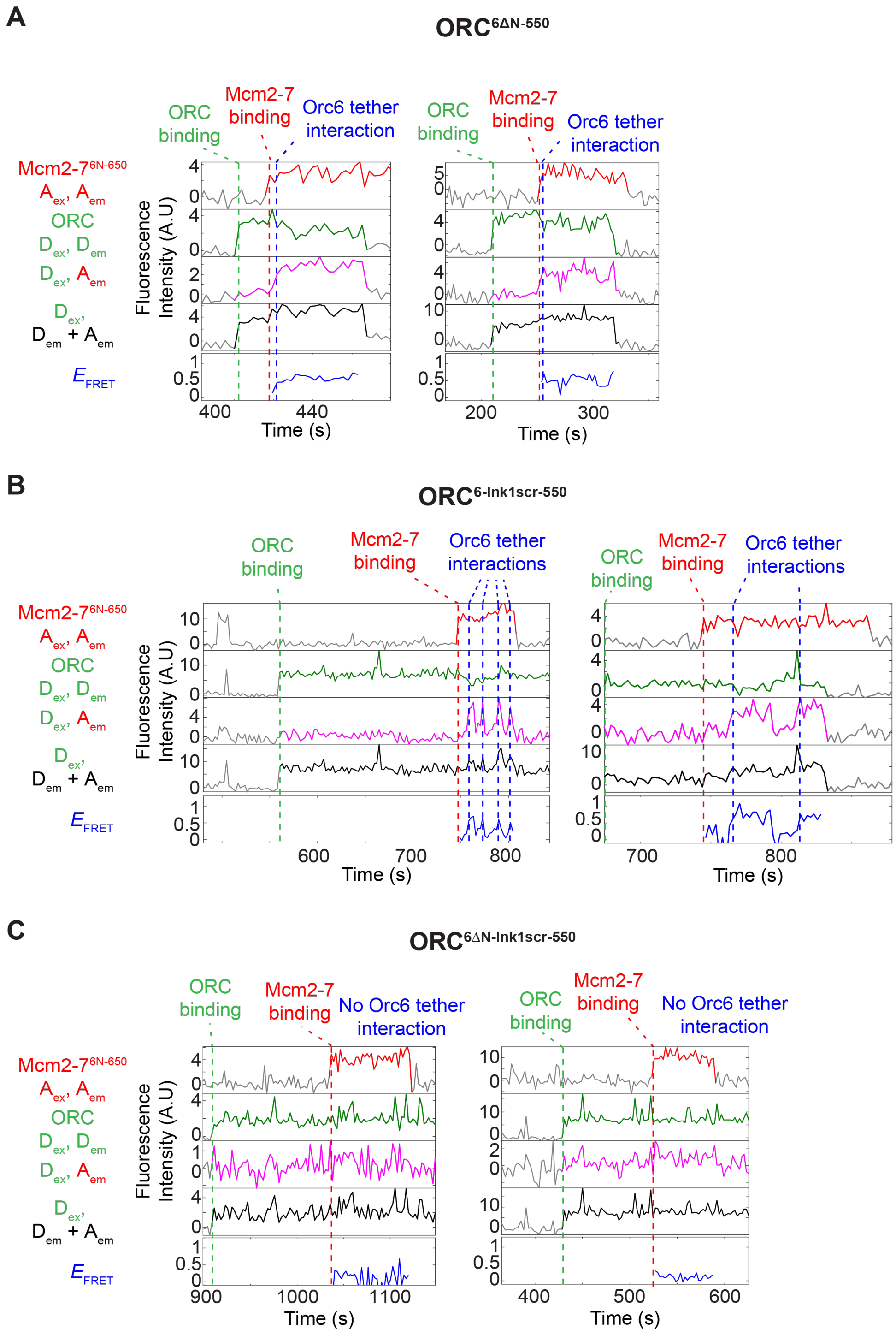

Figure S5: Representative single-DNA records from helicase-loading experiments described in Figure 2 using Mcm2-7$^{6N\text{-}650}$ and ORC$^{6\Delta N\text{-}550}$ (A), ORC$^{6\text{-}Ink1scr\text{-}550}$ (B), or ORC$^{6\Delta NInk1scr550}$. Records are plotted as in Figure 1C.

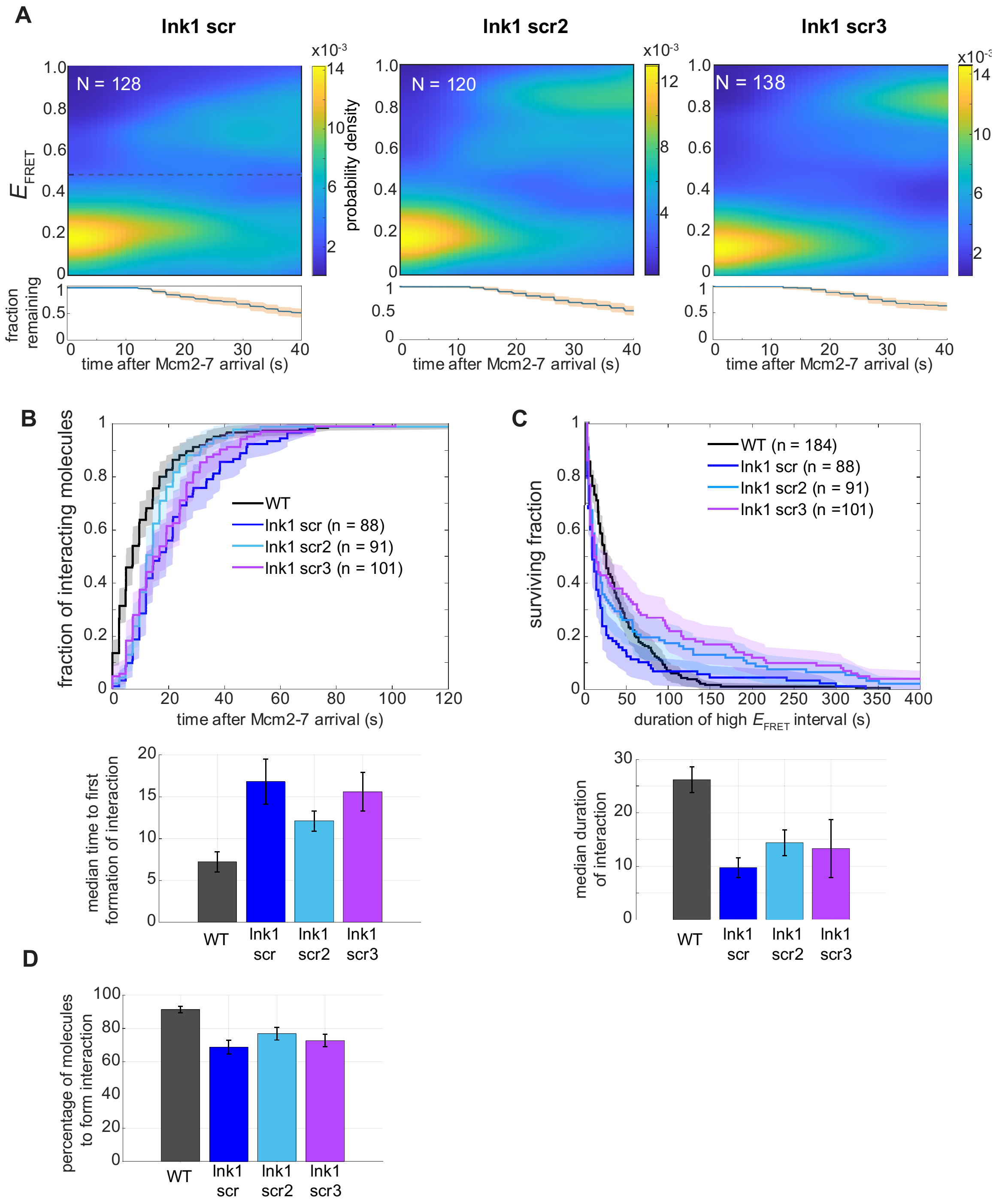

Figure S6: Different Orc6 Ink1 scrambled mutations have similar effects on Orc6 tether interactions.

A: Heat maps as described in Fig 2B using the mutant constructs: $ORC^{6\text{-Ink1scr-550}}$ (left), $ORC^{6\text{-Ink1scr2-550}}$ (middle), and $ORC^{6\text{-Ink1scr3-550}}$ (right).

Mutant Ink1 amino acid sequences are as follows:

Ink1 scr: ASEGKPTFEN GDVKEPSPLS VQRDPFESLQ KKFNPMPNTR EKLRSNNNSL RTSTSTGPR IVIQFKR
Ink1 scr2: LEPRVSPETF LRSNSNKFFK DALKKTPQSK NTRRGKQNS QNRPGIEEVT SEMFSIPDGN PLDVRPT
Ink1 scr3: GGSLRFTTEI NRPFKVNPPF NGPKESNTDR SSRTVASSD KRPNDKEKQL KTVEFPMPQS QLNERLI

B: Time to first formation of Orc6 tether interaction plotted relative to Mcm2-$7^{6N\text{-}650}$ arrival as in Fig 2C. Below: Bar graph showing median times (± S.E.) to reach high E_{FRET} state.

C: Cumulative survival curve of initial Orc6 tether interactions as in Fig 2D. Below: Bar graph showing median durations (± S.E.) of high E_{FRET} state for each mutant.

D: Percentage of molecules that formed the Orc6 tether interaction as in Fig 2E.

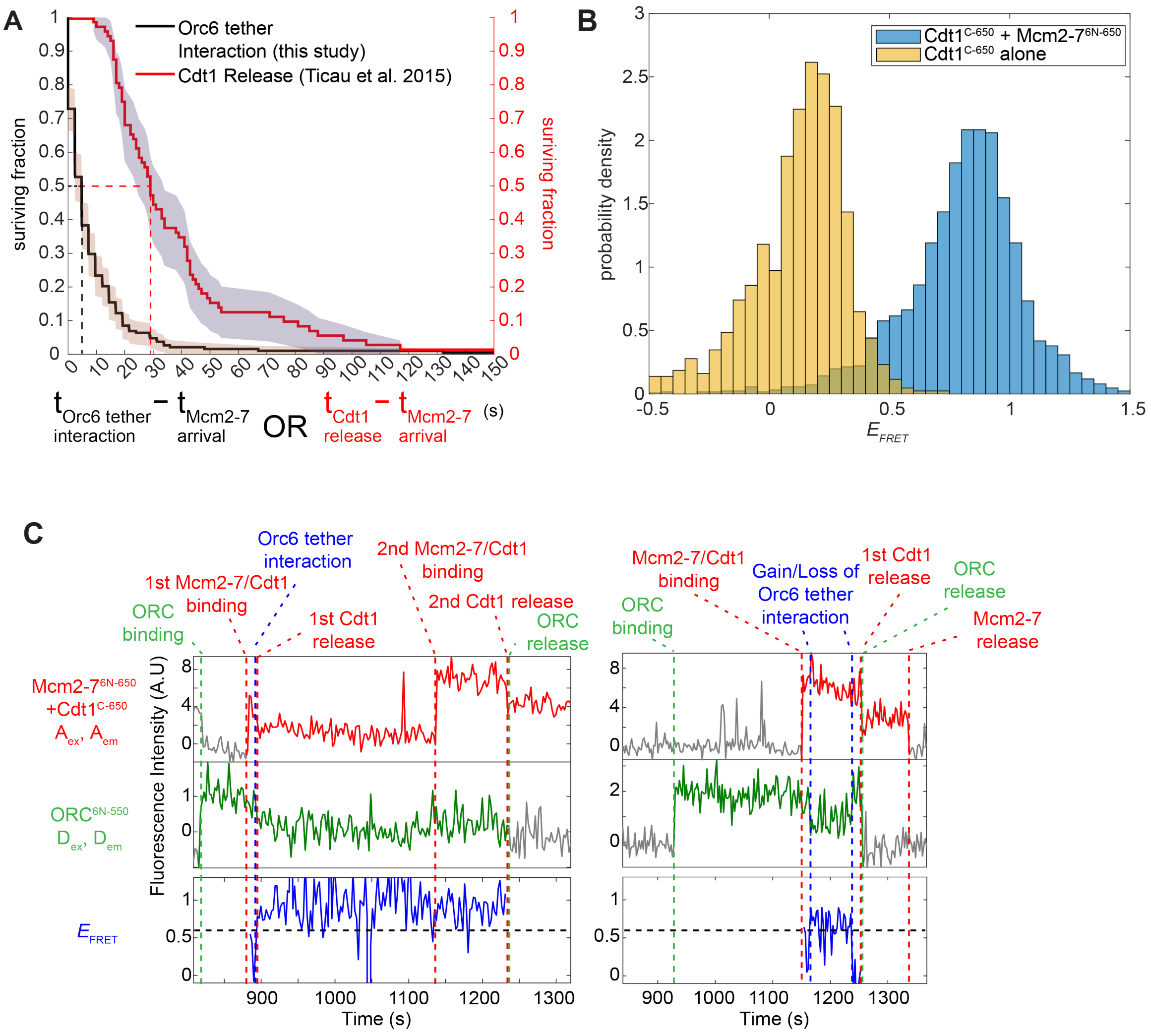

Figure S7: Timing of Orc6 tether interaction and Cdt1 release.

A: Survival plot of the fraction of molecules with Cdt1 (red) or that have yet to form the Orc6 tether interaction (black). All times are relative to arrival of first Mcm2-7 at the same DNA molecule. Shaded areas represent 95% CI. Dashed lines indicate median times for each dataset.

B: $Cdt1^{C\text{-}650}$ does not exhibit high E_{FRET} with $ORC^{6N\text{-}550}$ during helicase loading. Histogram plots of E_{FRET} values from $Mcm2\text{-}7^{6N\text{-}650}$ recruitment events during each frame of colocalization of ORC and Mcm2-7/Cdt1 to DNA. Yellow bars: $Cdt1^{C\text{-}650}$, $ORC^{6N\text{-}550}$ and unlabeled Mcm2-7 and Cdc6. Blue bars: $Cdt1^{C\text{-}650}$, $ORC^{6N\text{-}550}$, $Mcm2\text{-}7^{6N\text{-}650}$ and unlabeled Cdc6.

C: Additional traces of Orc6 tether interaction and Cdt1-association assay using $ORC^{6N\text{-}550}$, $Mcm2\text{-}7^{6N\text{-}650}$ and $Cdt1^{C\text{-}650}$. The right panel shows an example in which E_{FRET} is low at the time of 1st Cdt1 release and, consistent with a tether function, ORC release occurs rapidly after 1st Cdt1 release. Traces are arranged as described in Figure 3C.

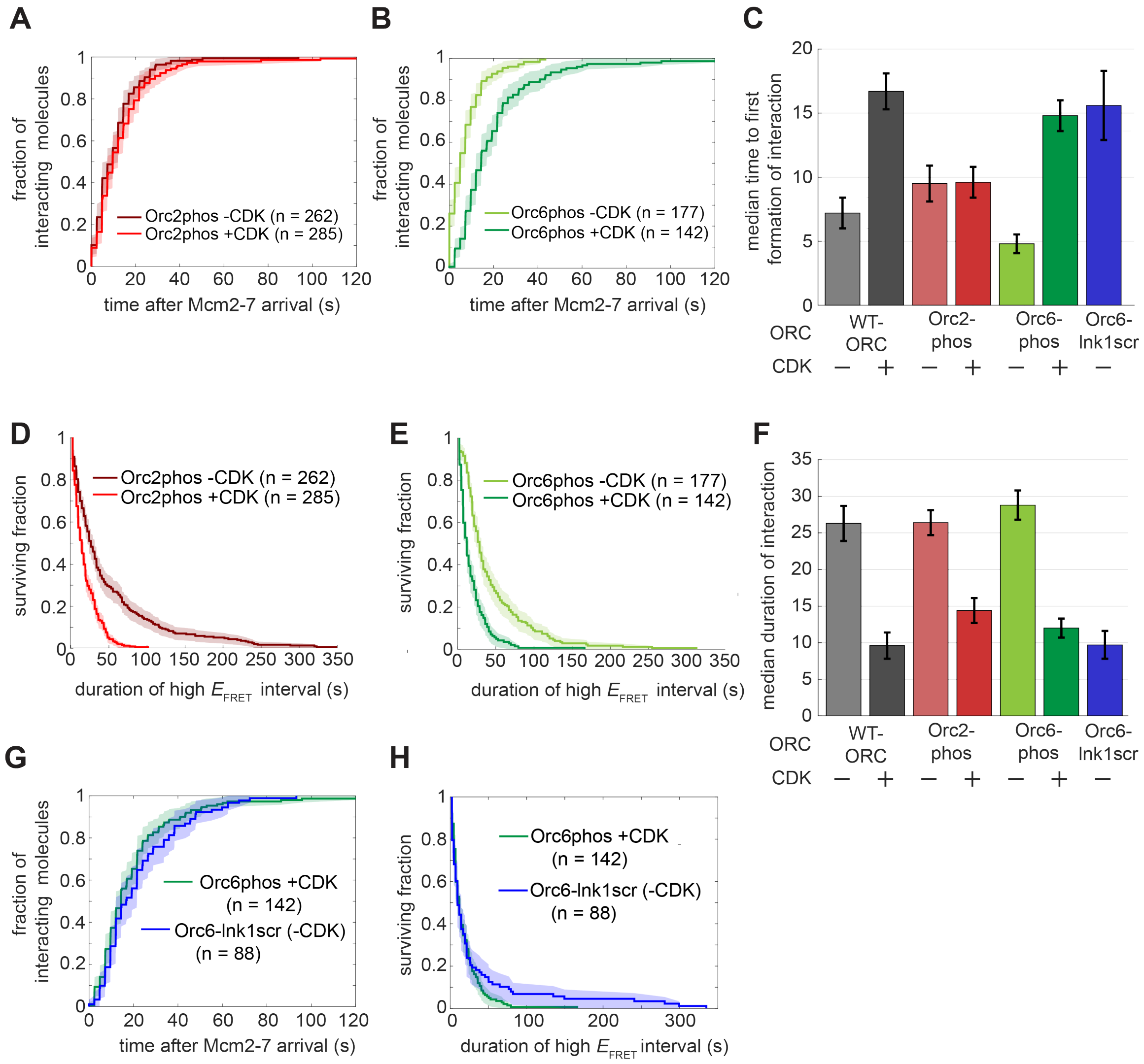

Figure S8: Phosphorylation of Orc6 alone inhibits median time to formation of Orc6 tether, but phosphorylation of either Orc2 or Orc6 impacts the stability of the Orc6 tether interaction.

A: Plot same as Figure 4C but using $ORC^{2phos\text{-}6N\text{-}550}$.

B: Plot same as Figure 4C but using $ORC^{6phos\text{-}6N\text{-}550}$.

C: Median time to first tether interaction formation with and without CDK modification for WT, $ORC^{2phos\text{-}6N\text{-}550}$, and $ORC^{6phos\text{-}6N\text{-}550}$. The time to first formation (± S.E.) for unmodified $ORC^{6\text{-}Ink1scr\text{-}650}$ (Figure 2C) is shown for comparison.

D: Plot same as Figure 4D but using $ORC^{2phos\text{-}6N\text{-}550}$.

E: Plot same as Figure 4D but using $ORC^{6phos\text{-}6N\text{-}550}$.

F: Median duration of first tether interaction with and without CDK modification for WT, $ORC^{2phos\text{-}6N\text{-}550}$, and $ORC^{6phos\text{-}6N\text{-}550}$. Median duration of first tether interaction (± S.E.) for unmodified $ORC^{6\text{-}Ink1scr\text{-}650}$ (Figure 2D) is shown for comparison.

G: Plot same as Figure 4C but now comparing $ORC^{6phos\text{-}6N\text{-}550}$ +CDK (green, same data as B) to $ORC^{6\text{-}Ink1scr\text{-}550}$ (blue, same data as Figure 2C).

H: Cumulative survival curve of initial Orc6 tether interactions for CDK-modified $ORC^{6phos\text{-}6N\text{-}550}$ (green) or unmodified $ORC^{6\text{-}Ink1scr\text{-}550}$ (blue). Shading represents 95% CI. Note, ~20% of $ORC^{6\text{-}Ink1scr\text{-}550}$ molecules retain the tether interaction for significantly longer times compared to $ORC^{6phos\text{-}6N\text{-}550}$ +CDK.

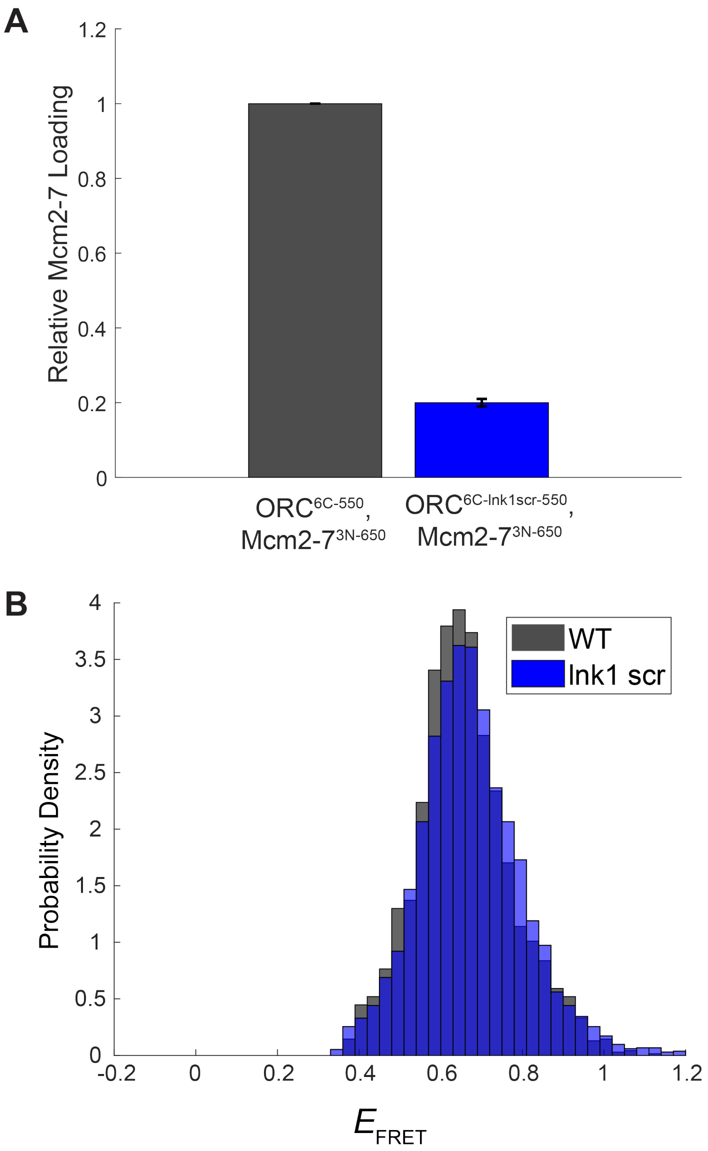

Figure S9: Further analysis of Orc6 Ink1 function.

A: ORC$^{6C\text{-}Ink1scr\text{-}550}$ is deficient in double-hexamer formation in ensemble helicase-loading assays. Ensemble helicase-loading assays were performed with ORC$^{6C\text{-}550}$ or ORC$^{6C\text{-}Ink1scr\text{-}550}$ and Mcm2-7$^{3N\text{-}650}$, along with unlabeled Cdt1 and Cdc6. Bars indicate the relative loading of salt-stable helicases. Errors are S.E.M. N = 2 for each reaction.

B: E_{FRET} values of MO complex for ORC$^{6C\text{-}550}$ and ORC$^{6C\text{-}Ink1scr\text{-}550}$ are similar. E_{FRET} values from each experiment (black: ORC$^{6C\text{-}550}$, blue: ORC$^{6C\text{-}Ink1scr\text{-}550}$) were computed and binned into low and high E_{FRET} states based on threshold determination (see Figure S2E-F, Methods). The resulting high E_{FRET} values were plotted as a histogram and normalized by probability density to compare the distribution of high E_{FRET} values.

Table S1. Fit parameters for E_{FRET} distributions*

ORC	Mcm2-7	Cdt1	Figure	A	μ_1	σ_1	μ_2	σ_2	N
$ORC^{6N\text{-}550}$	$Mcm2\text{-}7^{6N\text{-}650}$	WT	1	0.384	0.33	0.22	0.71	0.17	7613
$ORC^{6\Delta N\text{-}550}$	$Mcm2\text{-}7^{6N\text{-}650}$	WT	2	0.414	0.20	0.18	0.62	0.18	3226
$ORC^{6\text{-}lnk1scr\text{-}550}$	$Mcm2\text{-}7^{6N\text{-}650}$	WT	2	0.473	0.17	0.17	0.80	0.16	4239
$ORC^{6\Delta N\text{-}lnk1scr\text{-}550}$	$Mcm2\text{-}7^{6N\text{-}650}$	WT	2	0.991	0.11	0.12	0.57	0.30	3137
$ORC^{6N\text{-}550}$	$Mcm2\text{-}7^{6N\text{-}650}$	$Cdt1^{C\text{-}650}$	3	0.228	0.43	0.25	0.84	0.15	7680
$ORC^{6N\text{-}550}$ + CDK	$Mcm2\text{-}7^{6N\text{-}650}$	WT	4	0.776	0.14	0.14	0.59	0.14	4894
$ORC^{6C\text{-}550}$	$Mcm2\text{-}7^{3N\text{-}650}$	WT	5	0.503	0.11	0.13	0.65	0.13	5490
$ORC^{6C\text{-}lnk1scr\text{-}550}$	$Mcm2\text{-}7^{3N\text{-}650}$	WT	5	0.868	0.10	0.13	0.66	0.13	6089

*A, proportion of data in low E_{FRET} component; μ_1, mean of low E_{FRET} component; σ_1, SD of low E_{FRET} component; μ_2, mean of high E_{FRET} component; σ_2, SD of high E_{FRET} component; N = number of observations for fits.

Table S2. List of yeast strains used in this study

Strain Name	Construct	Genotype	Study
yDD57	ORC^{6N-550}	*ade2-1 trp1-1 leu2-3,112 his3-11,15 ura3-1 can1-100 bar1::hisG lys2::HisG pep4::unmarked URA3::pAZ96 (GAL1,10 FLAG-ORC1-opt, ORC2-opt) HIS3::pJF17 (ORC3-opt, ORC4-opt) TRP1::pDD48 (GAL1,10 ORC5, ORC6-107-S6)*	This study
yDD50	$Mcm2\text{-}7^{6N-650}$/Cdt1	*ade2-1 trp1-1 leu2-3,112 his3-11,15 ura3-1 can1-100 bar1::hisG lys2::HisG pep4::unmarked LYS2::pSKM002 (GAL1,10 MCM4, MCM5) TRP1::pDD36 (GAL1,10 UbSORT-mcm6∆103, MCM7) HIS3::pSKM004 (GAL1,10 MCM2, Flag-MCM3) URA3::pALS1 (GAL1,10 CDT1, GAL4)*	This study
ySG01	$Mcm2\text{-}7^{2C-650}$/Cdt1	*ade2-1 trp1-1 leu2-3,112 his3-11,15 ura3-1 can1-100 bar1::hisG lys2::HisG pep4::unmarked LYS2::pSKM002 (GAL1,10 MCM4, MCM5) TRP1::pSKM003 (GAL1,10 MCM6, MCM7) HIS3::pST048 (GAL1,10 MCM2C-LPETGG, Flag-MCM3) URA3::pALS1 (GAL1,10 CDT1, GAL4)*	(2)
yDD60	$Mcm2\text{-}7^{2scr-6N-650}$/Cdt1	*ade2-1 trp1-1 leu2-3,112 his3-11,15 ura3-1 can1-100 bar1::hisG lys2::HisG pep4::unmarked LYS2::pSKM002 (GAL1,10 MCM4, MCM5) TRP1::pSKM003 (GAL1,10 MCM6, MCM7) HIS3::pDD56 (GAL1,10 mcm2-348-*	This study

		365-scr, Flag-MCM3) URA3::pALS1 (GAL1,10 CDT1, GAL4)	
yAZ90	ORC$^{6\Delta N\text{-}550}$	*ade2-1 trp1-1 leu2-3,112 his3-11,15 ura3-1 can1-100 bar1::hisG lys2::HisG pep4::unmarked URA3::pAZ96 (GAL1,10 FLAG-ORC1-opt, ORC2-opt) HIS3::pJF17 (ORC3-opt, ORC4-opt) TRP1::pAZ87 (GAL1,10 ORC5, UbSORT-orc6ΔN)*	This study
yDD54	ORC$^{6\text{-lnk1scr-}550}$	*ade2-1 trp1-1 leu2-3,112 his3-11,15 ura3-1 can1-100 bar1::hisG lys2::HisG pep4::unmarked URA3::pAZ96 (GAL1,10 FLAG-ORC1-opt, ORC2-opt) HIS3::pJF17 (ORC3-opt, ORC4-opt) TRP1::pDD53 (GAL1,10 ORC5, orc6-107-S6-lnk1scr)*	This study
yDD52	ORC$^{6\Delta N\text{-lnk1scr-}550}$	*ade2-1 trp1-1 leu2-3,112 his3-11,15 ura3-1 can1-100 bar1::hisG lys2::HisG pep4::unmarked URA3::pAZ96 (GAL1,10 FLAG-ORC1-opt, ORC2-opt) HIS3::pJF17 (ORC3-opt, ORC4-opt) TRP1::pDD49 (GAL1,10 ORC5, UbSORT-orc6ΔN-lnk1scr)*	This study
ySC136	ORC6 swapper strain	*ade2-1 ura3-1 his3-11,15 trp1-1 leu2-3,112 can1-100 lys2::hisG bar1::hisG orc6::KanMX MATa pSPB66 (ORC6, URA3)*	(3)
ySC154	Orc3pro-ORC6	*ade2-1 ura3-1 his3-11,15 trp1-1 leu2-3,112 can1-100 lys2::hisG bar1::hisG orc6::KanMX MATa pSPB66 (ORC6, URA3) TRP1::pRS404-Orc3p-ORC6*	(3)

yDD70	Orc3pro-*orc6ΔN*	*ade2-1 ura3-1 his3-11,15 trp1-1 leu2-3,112 can1-100 lys2::hisG bar1::hisG orc6::KanMX MATa pSPB66 (ORC6, URA3) TRP1::pDD73 (Orc3p-orc6ΔN)*	This study
yDD71	Orc3pro-*orc6-lnk1scr*	*ade2-1 ura3-1 his3-11,15 trp1-1 leu2-3,112 can1-100 lys2::hisG bar1::hisG orc6::KanMX MATa pSPB66 (ORC6, URA3) TRP1::pDD74 (Orc3p-orc6-lnk1scr)*	This study
yDD72	Orc3pro-*orc6ΔN-lnk1scr*	*ade2-1 ura3-1 his3-11,15 trp1-1 leu2-3,112 can1-100 lys2::hisG bar1::hisG orc6::KanMX MATa pSPB66 (ORC6, URA3) TRP1::pDD75 (Orc3p-orc6ΔN-lnk1scr)*	This study
yDD75	$ORC^{6\text{-new-lnk1scr-1-550}}$	*ade2-1 trp1-1 leu2-3,112 his3-11,15 ura3-1 can1-100 bar1::hisG lys2::HisG pep4::unmarked URA3::pAZ96 (GAL1,10 FLAG-ORC1-opt, ORC2-opt) HIS3::pJF17 (ORC3-opt, ORC4-opt) TRP1::pDD76 (GAL1,10 ORC5, orc6-107-S6-new-lnk1scr-1)*	This study
yDD76	$ORC^{6\text{-new-lnk1scr-2-550}}$	*ade2-1 trp1-1 leu2-3,112 his3-11,15 ura3-1 can1-100 bar1::hisG lys2::HisG pep4::unmarked URA3::pAZ96 (GAL1,10 FLAG-ORC1-opt, ORC2-opt) HIS3::pJF17 (ORC3-opt, ORC4-opt) TRP1::pDD77 (GAL1,10 ORC5, orc6-107-S6-new-lnk1scr-2)*	This study
ASY1055.1	MCM2 swapper strain	*MATa ade2-1 ura3-11 his3-11,15 leu2-3,12 can-100 trp1-1 mcm2::hisG [pMCM5-MCM2::URA3]*	(4)
yDD77	Mcm5p-*mcm2-Δ348-365*	*ade2-1 ura3-1 his3-11,15 trp1-1 leu2-3,112 can1-100 lys2::hisG bar1::hisG orc6::KanMX MATa pMcm5pro-*	This study

		Mcm2(MCM2, URA3) TRP1::pDD57 (Mcm5p-mcm2-Δ348-365)	
yDD78	Mcm5p-*mcm2-348-365-GGS*	*ade2-1 ura3-1 his3-11,15 trp1-1 leu2-3,112 can1-100 lys2::hisG bar1::hisG orc6::KanMX MATa pMcm5pro-Mcm2(MCM2, URA3) TRP1::pDD58 (Mcm5p-mcm2-348-365-GGS)*	This study
yDD79	Mcm5p-*mcm2-348-365-scr*	*ade2-1 ura3-1 his3-11,15 trp1-1 leu2-3,112 can1-100 lys2::hisG bar1::hisG orc6::KanMX MATa pMcm5pro-Mcm2(MCM2, URA3) TRP1::pDD57 (Mcm5p-mcm2-348-365-scr)*	This study
yDD46	Mcm2-7$^{6N\text{-}650}$ (no Cdt1)	*ade2-1 trp1-1 leu2-3,112 his3-11,15 ura3-1 can1-100 bar1::hisG lys2::HisG pep4::unmarked LYS2::pSKM002 (GAL1,10 MCM4, MCM5) TRP1::pDD36 (GAL1,10 UbSORT-mcm6Δ103, MCM7) HIS3::pSKM004 (GAL1,10 MCM2, Flag-MCM3)*	This study
ySG46	Cdt1$^{C\text{-}650}$	*ade2-1 trp1-1 leu2-3,112 his3-11,15 ura3-1 can1-100 bar1::hisG lys2::HisG pep4::unmarked URA3::pSG26(GAL1,10-3xFLAG-Cdt1-C-LPETGG)*	(2)
ySK119	CDK	*ade2-1 trp1-1 leu2-3,112 his3-11,15 ura3-1 can1-100 bar1::HisG lys2::HisG pep4::unmarked URA3::GAL1,10 Δ2-95-CLB5-Flag CDC28-His*	(5)
yDD73	Orc2phos	*ade2-1 trp1-1 leu2-3,112 his3-11,15 ura3-1 can1-100 bar1::hisG lys2::HisG pep4::unmarked URA3::pAZ96 (GAL1,10*	This study

		FLAG-ORC1-opt, ORC2) HIS3::pJF17 (ORC3-opt, ORC4-opt) TRP1::pDD64 (GAL1,10 ORC5, orc6-107-S6-4A)	
yDD74	Orc6phos	*ade2-1 trp1-1 leu2-3,112 his3-11,15 ura3-1 can1-100 bar1::hisG lys2::HisG pep4::unmarked URA3::pSG50 (GAL1,10 FLAG-ORC1-opt, orc2-6A) HIS3::pJF17 (ORC3-opt, ORC4-opt) TRP1::pDD48 (GAL1,10 ORC5, ORC6-107-S6)*	This study
ySG39	ORC^{6C-550}	*ade2-1 trp1-1 leu2-3,112 his3-11,15 ura3-1 can1-100 bar1::hisG lys2::HisG pep4::unmarked URA3::pJF19 (GAL1,10 CBP-ORC1-opt, ORC2-opt) HIS3::pJF17 (ORC3-opt, ORC4-opt) TRP1::pAZ63 (GAL1,10 ORC5-opt, ORC6-C-LPETGG)*	(2)
ySG24	$Mcm2\text{-}7^{3N-650}$	*ade2-1 trp1-1 leu2-3,112 his3-11,15 ura3-1 can1-100 bar1::hisG lys2::HisG pep4::unmarked LYS2::pSKM002 (GAL1,10 MCM4, MCM5) TRP1::pSKM003 (GAL1,10 MCM6, MCM7) HIS3::pSG13 (GAL1,10 MCM2, Flag-TEV-GG-MCM3)*	(2)
ySG54	$ORC^{6C-lnk1scr-550}$	*ade2-1 trp1-1 leu2-3,112 his3-11,15 ura3-1 can1-100 bar1::hisG lys2::HisG pep4::unmarked URA3::pAZ96 (GAL1,10 FLAG-ORC1-opt, ORC2-opt) HIS3::pJF17 (ORC3-opt, ORC4-opt) TRP1::pSG46 (GAL1,10 ORC5-opt, orc6-lnk1scr-C-LPETGG)*	This study

Table S3: List of plasmids used in this study

Name	Construct	Description	Study
pJF17	ORC3 + ORC4	*GAL1,10 ORC3-opt, ORC4-opt*	(6)
pJF19	CBP-ORC1 + ORC2	*GAL1,10 CBP-ORC1-opt, ORC2-opt*	(6)
pAZ96	FLAG-ORC1 + ORC2	*GAL1,10 FLAG-ORC1-opt, ORC2-opt*	(2)
pSKM002	MCM4 + MCM5	*GAL1,10 MCM4, MCM5*	(7)
pSKM003	MCM6 + MCM7	*GAL1,10 MCM6, MCM7*	(7)
pSKM004	MCM2 + FLAG-MCM3	*GAL1,10 MCM2, Flag-MCM3*	(7)
pSKM033	FLAG-Cdc6	*pGEX- GST-PP-FLAG-Cdc6*	(7)
pALS1	CDT1 + GAL4	*GAL1,10 CDT1, GAL4*	(7)
pDD48	ORC5 + ORC6-107-S6	*GAL1,10 ORC5, ORC6-107-S6*	This study
pDD36	UbSORT-*mcm6Δ103* + MCM7	*GAL1,10 UbSORT-mcm6Δ103, MCM7*	This study
pST048	MCM2-C-LPETGG + FLAG-MCM3	*GAL1,10 MCM2C-LPETGG, Flag-MCM3*	(2)
pDD56	*mcm2-348-365-scr* + FLAG-MCM3	*GAL1,10 mcm2-348-365-scr, Flag-MCM3*	This study
pAZ87	ORC5 + UbSORT-*orc6ΔN*	*GAL1,10 ORC5, UbSORT-orc6ΔN*	This study
pDD53	ORC5 + *orc6-107-S6-lnk1scr*	*GAL1,10 ORC5, orc6-107-S6-lnk1scr*	This study
pDD49	ORC5 + UbSORT-*orc6ΔN-lnk1scr*	*GAL1,10 ORC5, UbSORT-orc6ΔN-lnk1scr*	This study
pSPB66	ORC6, URA3	*ORC6, URA3*	(3)
pRS404-Orc3p-ORC6	Orc3p-ORC6	*Orc3pro-ORC6*	(3)
pDD73	Orc3p-*orc6ΔN*	*Orc3pro-orc6ΔN*	This study
pDD74	Orc3p-*orc6-lnk1scr*	*Orc3pro-orc6-lnk1scr*	This study
pDD75	Orc3p-*orc6ΔN-lnk1scr*	*Orc3pro-orc6ΔN-lnk1scr*	This study
pDD57	Mcm5p-*mcm2-Δ348-365*	*Mcm5pro-mcm2-Δ348-365*	This study
pDD58	Mcm5p-*mcm2-348-365-GGS*	*Mcm5pro-mcm2-348-365-GGS*	This study
pDD59	Mcm5p-*mcm2-348-365-scr*	*Mcm5pro-mcm2-348-365-scr*	This study
pDD76	Orc6-new-lnk1scr-1	*GAL1,10 ORC5, orc6-107-S6-new-lnk1scr-1*	This study
pDD77	Orc6-new-lnk1scr-2	*GAL1,10 ORC5, orc6-107-S6-new-lnk1scr-2*	This study
pSG26	Cdt1-C-SORT	*GAL1,10-3xFLAG-Cdt1-C-LPETGG*	(2)
pRS306 deltN95 Clb5-Flag Cdc28-His	Clb5 + Cdc28	*GAL1,10 Δ2-95-CLB5-Flag CDC28-His*	(5)
pGEX-Sic1	Sic1	*pGEX-Sic1*	(5)
pDD64	ORC5 + *orc6-107-S6-4A*	*GAL1,10 ORC5, orc6-107-S6-4A*	This study
pSG50	FLAG-ORC1 + *orc2-6A*	*GAL1,10 FLAG-ORC1-opt, orc2-6A*	(8)

pAZ63	ORC5 + ORC6-C-LPETGG	*GAL1,10 ORC5-opt, ORC6-C-LPETGG*	(2)
pSG13	MCM2 + FLAG-TEV-GG-MCM3	*GAL1,10 MCM2, Flag-TEV-GG-MCM3*	(2)
pSG46	ORC5 + *orc6-lnk1scr-*C-LPETGG	*GAL1,10 ORC5-opt, orc6-lnk1scr-C-LPETGG*	This study

SI Methods

FRET data analysis

Apparent E_{FRET} calculations performed as described (2). To determine identity of low and high E_{FRET} states, the E_{FRET} data for ORC and Mcm2-7$^{6N\text{-}650}$ interactions (experiments in Figure S2) were selected from two time intervals after first Mcm2-7$^{6N\text{-}650}$ arrival for each experiment: 0 to 5 s and >5 s. E_{FRET} values from -0.2 to 1.2 (which constitute >97% of observations) from each interval were independently fit to the two-component Gaussian mixture probability density function:

$$\frac{1}{\sqrt{2\pi}}\left\{\frac{A}{\sigma_1}\exp\left[-\frac{(E_{FRET}-\mu_1)^2}{2{\sigma_1}^2}\right]+\frac{(1-A)}{\sigma_2}\exp\left[-\frac{(E_{FRET}-\mu_2)^2}{2{\sigma_2}^2}\right]\right\}$$

where A is the fractional amplitude of the low E_{FRET} component, $\mu_1 < \mu_2$ are the mean E_{FRET} values of the low and high components, and σ_1, σ_2 are the SDs of the low and high E_{FRET} components. SEs of the fit parameters were computed by bootstrapping (1,000 samples). The E_{FRET} threshold value used to differentiate between the low and high E_{FRET} state was defined as the crossing point of the two components of the gaussian fit for each dataset. An E_{FRET} transition from low to high was considered to have taken place once the E_{FRET} value crossed the threshold for two consecutive frames. An E_{FRET} transition from high to low was considered to have taken place once the E_{FRET} value crossed below the threshold for two consecutive frames. Median time to first formation of interaction (Figure S1E, 2C, 4C, S7C) was determined by taking the median time at which the high E_{FRET} state was reached for all molecules that successfully made a transition from low to high E_{FRET}. Median duration of interaction (Figure S1F, 2D, 4D, S7F) was determined by taking the

median time of the duration of the high E_{FRET} states for all molecules that made a transition from low to high E_{FRET} during the experiment.

E_{FRET} heat maps

E_{FRET} heat maps were generated as described (9). In brief, heat maps were constructed using MATLAB code adapted from https://github.com/gelles-brandeis/jganalyze. The code uses two-dimensional kernel density estimation, with a normal kernel function (standard deviation of time axis = 5s and E_{FRET} axis 0.05) to generate a heat map of E_{FRET} values versus time (0-40 seconds after first Mcm2-7 arrival). Each vertical slice represents the distribution of E_{FRET} values at that particular time interval. The time axis has a resolution of 1 second and the E_{FRET} axis has a resolution of 0.005. Normalization was performed such that the density estimate at each time slice integrates to 1.

Source data availability

Source data for the single-molecule experiments are provided as Matlab "intervals" files that can be read and manipulated by the program imscroll (https://github.com/gellesbrandeis/CoSMoS_Analysis)(10). The source data are archived at doi: https://doi.org/10.5281/zenodo.15231065. (11)

SI References

1. G. Erdős, Z. Dosztányi, AIUPred: combining energy estimation with deep learning for the enhanced prediction of protein disorder. *Nucleic Acids Res.* **52**, W176–W181 (2024).
2. S. Gupta, L. J. Friedman, J. Gelles, S. P. Bell, A helicase-tethered ORC flip enables bidirectional helicase loading. *eLife* **10**, e74282 (2021).
3. S. Chen, S. P. Bell, CDK prevents Mcm2–7 helicase loading by inhibiting Cdt1 interaction with Orc6. *Genes Dev.* **25**, 363–372 (2011).
4. A. Schwacha, S. P. Bell, Interactions between Two Catalytically Distinct MCM Subgroups Are Essential for Coordinated ATP Hydrolysis and DNA Replication. *Mol. Cell* **8**, 1093–1104 (2001).
5. R. C. Heller, *et al.*, Eukaryotic Origin-Dependent DNA Replication In Vitro Reveals Sequential Action of DDK and S-CDK Kinases. *Cell* **146**, 80–91 (2011).

6. J. Frigola, D. Remus, A. Mehanna, J. F. X. Diffley, ATPase-dependent quality control of DNA replication origin licensing. *Nature* **495**, 339–343 (2013).
7. S. Kang, M. D. Warner, S. P. Bell, Multiple Functions for Mcm2–7 ATPase Motifs during Replication Initiation. *Mol. Cell* **55**, 655–665 (2014).
8. A. L. Amasino, S. Gupta, L. J. Friedman, J. Gelles, S. P. Bell, Regulation of replication origin licensing by ORC phosphorylation reveals a two-step mechanism for Mcm2-7 ring closing. *Proc. Natl. Acad. Sci.* **120**, e2221484120 (2023).
9. A. Zhang, L. J. Friedman, J. Gelles, S. P. Bell, Changing protein–DNA interactions promote ORC binding-site exchange during replication origin licensing. *Proc. Natl. Acad. Sci.* **120**, e2305556120 (2023).
10. L. J. Friedman, J. Gelles, Multi-wavelength single-molecule fluorescence analysis of transcription mechanisms. *Methods* **86**, 27–36 (2015).
11. D. Driscoll, An Orc6 tether mediates ORC binding site switching during replication origin licensing. Zenodo. https://doi.org/10.5281/zenodo.15231065.
